# Screening of *Mycobacterium avium* subsp. *paratuberculosis* mutants for attenuation in a bovine monocyte-derived macrophage model

**DOI:** 10.3389/fcimb.2014.00087

**Published:** 2014-06-30

**Authors:** Elise A. Lamont, Adel M. Talaat, Paul M. Coussens, John P. Bannantine, Yrjo T. Grohn, Robab Katani, Ling-ling Li, Vivek Kapur, Srinand Sreevatsan

**Affiliations:** ^1^Department of Veterinary Population Medicine, University of MinnesotaSt. Paul, MN, USA; ^2^Department of Pathobiological Sciences, University of WisconsinMadison, WI, USA; ^3^Department of Animal Sciences, Michigan State UniversityEast Lansing, MI, USA; ^4^Agricultural Research Service, National Animal Disease Center, United States Department of AgricultureAmes, IA, USA; ^5^College of Veterinary Medicine, Population Medicine and Diagnostic Sciences, Cornell UniversityIthaca, NY, USA; ^6^Department Veterinary and Biomedical Sciences, Penn State UniversityState College, PA, USA; ^7^Department of Veterinary Biomedical Sciences, University of MinnesotaSt. Paul, MN, USA

**Keywords:** *Mycobacterium avium* subsp. *paratuberculosis*, Johne's disease, vaccine, macrophage, mutant, transposon mutagenesis, survival

## Abstract

Vaccination remains a major tool for prevention and progression of Johne's disease, a chronic enteritis of ruminants worldwide. Currently there is only one licensed vaccine within the United States and two vaccines licensed internationally against Johne's disease. All licensed vaccines reduce fecal shedding of *Mycobacterium avium* subsp. *paratuberculosis* (MAP) and delay disease progression. However, there are no available vaccines that prevent disease onset. A joint effort by the Johne's Disease Integrated Program (JDIP), a USDA-funded consortium, and USDA—APHIS/VS sought to identify transposon insertion mutant strains as vaccine candidates in part of a three phase study. The focus of the Phase I study was to evaluate MAP mutant attenuation in a well-defined *in vitro* bovine monocyte-derived macrophage (MDM) model. Attenuation was determined by colony forming unit (CFUs) counts and slope estimates. Based on CFU counts alone, the MDM model did not identify any mutant that significantly differed from the wild-type control, MAP K-10. Slope estimates using mixed models approach identified six mutants as being attenuated. These were enrolled in protection studies involving murine and baby goat vaccination-challenge models. MDM based approach identified trends in attenuation but this did not correlate with protection in a natural host model. These results suggest the need for alternative strategies for Johne's disease vaccine candidate screening and evaluation.

## Introduction

Vaccination is arguably the most successful story in medical research that has improved the longevity and quality of life for both humans and animals (Koff et al., 2013). Currently there is one licensed vaccine for Johne's disease, a chronic enteritis of ruminants worldwide caused by *Mycobacterium avium* subsp. *paratuberculosis* (MAP), within the United States (Patton, 2011). Mycopar (Boehringer, Ingelheim, Ridgefield, CT, USA) is a heat-killed bacterin of *Mycobacterium avium* mixed with an oil adjuvant. Other vaccine formulations available internationally, Gudair (Zoetis, Inc.) and Silirum (Pfizer), are composed of heat inactivated MAP strain 316F (for use in ovine paratuberculosis) and a heat killed strain of MAP (for use in bovine paratuberculosis), respectively (Reddacliff et al., 2006; Stringer et al., 2013). All licensed vaccines reduce fecal shedding of MAP but fail to produce protection from initial infection and sterilizing immunity in ruminants (Chiodini, 1993; Reddacliff et al., 2006; Rosseels and Huygen, 2008; Huttner et al., 2012) Furthermore, these vaccine formulations frequently result in granulomas and/or lesions at the site of injection as well as antibody responses that cross-react with other pathogenic mycobacteria, and does not reliably differentiate vaccinated from infected animals, creating difficulties in disease detection (Mackintosh et al., 2005; Eppleston and Windsor, 2007; Stabel et al., 2011; Thomsen et al., 2012; Hines et al., 2014). Due to undesirable consequences of vaccination, which includes injection site reactions and inference with TB diagnostics, the United States limits Mycopar to herds that have a high MAP prevalence or those that have limited resources (financial, facility, etc.) to achieve Johne's disease control program standards (Usda-Aphis, 2010).

Despite the risk of potential immunization sequelae, several studies indicate that vaccination is a necessary component for Johne's disease prevention (Kormendy, 1994; Lu et al., 2013). In a stochastic simulation model of MAP infection and vaccination, Lu et al. showed that effective vaccination of animals reduced MAP prevalence to 0.44 and 0.19 at a 5 and 10 year follow-up period, respectively (Lu et al., 2013). It is important to note that the authors also concluded that a small chance (<0.15) of MAP persistence due to vertical transmission exists (Lu et al., 2013). Therefore, it is likely that Johne's disease eradication must encompass vaccination under the umbrella of a well-defined control program that includes standard management practices for rapid detection, hygiene, separation of newborns from adults, and culling of infected animals (Ferrouillet et al., 2009; Espejo et al., 2012).

The need for an improved Johne's disease vaccine within management programs prompted the implementation of a three-phase vaccine candidate evaluation that was administered by the Johne's Disease Integrated Program (JDIP). This vaccine project consisted of three blinded studies: Phase I involved the screening of 22 submitted strains of MAP, carrying deletions at critical housekeeping or putative virulence genes (herein collectively referred to as MAP mutants), for attenuation in a well-defined bovine monocyte-derived macrophage (MDM) model, Phase II involved identified attenuated mutants from Phase I evaluation in a mouse vaccination-challenge model, and Phase III involved the evaluation of selected mutants from Phase II in a goat challenge model to evaluate protection in a ruminant host, which is the target species. We herein report results from the Phase I study. Mutant MAP strains classified as candidates for further testing were defined as those displaying significant attenuation, particularly based on slope trends indicative of total bacterial decay over the first 48-h post infection.

## Materials and methods

### Ethics statement

All animal work was conducted in accordance with the recommendations in the institutional guidelines and approved animal care and use committee (IACUC) protocols at the University of Minnesota (approval number 1106A01161). All other experiments were carried out in accordance with the University of Minnesota's Institutional Biosafety Committee (IBC) approved protocol number 0806H36901.

### Study design

All experiments were conducted in a double-blind design. The attenuated mutant strains were cultured, coded, and shipped to the testing labs by the coordinating team at Penn State University. At the end of the study, the MDM survival data of strains was decoded and analyzed by the JDIP Statistical Core at Cornell University.

### Purification of bovine monocyte-derived macrophages (MDMs)

Purification of bovine MDMs was conducted using a published protocol (Janagama et al., 2006). Briefly, whole blood was collected from the jugular vein into gas sterilized vacuum buckets containing ACD or CPDA anticoagulant. The blood was centrifuged at 2000 × g for 20 min at room temperature (RT) and buffy coats were harvested and diluted 1:10 in PBS before separation over a 58% percol gradient. After centrifugation of the percol gradient at 400 × g for 30 min at RT, the mononuclear cells were collected using a sterile transfer pipette into a 20-ml new tube. Three PBS washes were performed on cells to remove remaining percol. After the last PBS wash, the cell pellet was resuspended in RPMI 1640 medium (Gibco, Invitrogen, Carlsbad, CA) with 20% autologous serum in Teflon wells. Cell suspensions were incubated inside a humidified chamber containing 5% CO_2_ at 37°C and allowed to differentiate for 4 days before they were seeded only cell culture flasks (day 5). Cell culture medium was changed every 2–3 days.

### Bacterial cell culture

MAP mutant strains submitted by multiple investigators were received at Pennsylvania State University and cultured in Middlebrook (MB) 7H9 supplemented with 10% glycerol, 1% oleic acid-dextrose-catalase (OADC) or ADC, 0.05% Tween-80, cycloheximide (1.0 g/L), mycobactin J (2 mg/mL) (Allied Monitor, and appropriate antibiotics at 37°C at 120 rpm. Growth was determined on a weekly basis by recording culture absorbance (O.D._600 nm_) after several passages through G25 and G27 needles. Upon reaching an O.D._600 nm_ of 0.5, cultures were tested for contamination by the absence of both growth on Brain-Heart Infusion (BHI) and blood agar for 48 h and *M. avium* specific PCR not present in MAP (primer sequences: F 5'-aaacccaagcagagaaacga; R 5'-gcgtcaatcaaagcctgttc). MAP mutant strains were blinded and shipped to testing laboratories, then used to infect MDMs immediately upon receipt. Colony counts were determined after shipment by plating serial dilutions (10^−4^ through 10^−7^) of each culture (in triplicate) on MB7H9 agar plates and incubating for 4–6 weeks. Baseline CFUs were calculated based on averages obtained from triplicate plates of 10^−5^ and 10^−6^ dilutions.

### Map invasion assay

Unless stated otherwise, all incubations were conducted in a 37°C humidified chamber containing 5% CO_2_. Bovine MDMs were counted via trypan blue exclusion, seeded at 2.0 × 10^6^ cells in T-25 culture flasks, and allowed to adhere for 2 h in the humidified chamber (5% CO_2_ at 37°C). Non-adherent cells were removed by washing flasks three times in 1× PBS. RPMI 1640 supplemented with 2% autologous serum (infection medium) was added to each flask prior to MAP invasion. MAP cultures were pelleted at 8000 × g for 10 min, washed thrice using 1× PBS, resuspended in infection medium (MOI 10:1), and vortex mixed for 5 min. MAP cells were repeatedly drawn through a 21-gauge needle to dissociate bacterial clumps. Cells were incubated at 37°C for 5 min to sediment any remaining clumps and only the upper 2/3 volume was used for the invasion assay. MDMs were infected with MAP for 2 h, washed thrice with 1× D-PBS to remove non-adherent bacteria, and recovered in fresh infection medium for the following time points: 3, 24, 48, 96, and 168 h. Time points were selected upon well-established steps in MAP invasion, gene and protein expression, and adaptation to the intracellular environment (Murphy et al., 2006; Scandurra et al., 2009, 2010; Lamont and Sreevatsan, 2010; Ghosh et al., 2013). Upon completion of time points, MDMs were washed thrice in 1× D-PBS and incubated with warmed 0.01% Triton X-100 in PBS for 5 min at RT. The lysate was subjected to differential centrifugation at 388 × g and 8000 × g for 5 min each to remove MDMs and pellet MAP, respectively. MAP pellets were washed thrice in 1× PBS at 8000 × g for 5 min to remove any remaining detergent, and resuspended in 1.0 mL of 1× PBS. All time points were conducted in triplicate. During the infection time course, medium was replaced every 2 days. Recovered MAP cells were serially diluted in 1× PBS and plated on MB7H9 agar supplemented with 10% OADC, mycobactin J (2 mg/mL), and appropriate antibiotics (kanamycin or hygromycin). All plates were incubated at 37°C for 6–8 weeks. *IS900* colony PCR was performed on randomly selected colonies from each dilution to confirm purity as described (Bull et al., 2000). All dilutions were plated in duplicate.

### Statistical analysis performed by the university of minnesota

CFU numbers from MAP mutant strains were normalized against the MAP K-10 starting inoculum in order to achieve direct comparisons at each post infection time point. Results were plotted and analyzed using One-Way analysis of variance (ANOVA) with Bonferroni correction in GraphPad Prism software (GraphPad Software, La Jolla, CA). *P* values of less than 0.05 were considered as statistically significant. Analysis is reflective of CFU counts from the University of Minnesota.

### Statistical analysis performed by JDIP core

CFU numbers were submitted to the JDIP core. CFUs in bovine MDMs measured from different time points and the change (i.e., slope) between 3 and 48 h was calculated to compare mutants, as follows. CFU measurements were not normalized to the standard type inoculum. The natural logarithm was applied to mean CFUs/mL (calculated for each time point, cow and strain). Slope estimates were calculated by subtracting the (natural logarithm of the mean CFU/ml count at 3 h) from the (natural logarithm of the mean CFU/ml count at 48 h) and dividing by 45 (=48–3). Sixty-nine observations (one for each combination of university, strain and cow) were recorded and analyzed using PROC GLIMMIX ver. 9.2 in SAS (SAS Institute Inc., Cary, NC) using a linear regression model. The outcome variable was the slope estimate described above. These slope estimates were assumed to follow a normal distribution. The independent variable strain was a fixed effect, with 17 levels. University was a random effect with 2 levels. Final analysis included observations recorded from both the University of Minnesota and the University of Wisconsin-Madison.

## Results

### Map mutant competence in an *in vitro* primary monocyte-derived macrophage model

**Figure 1:**
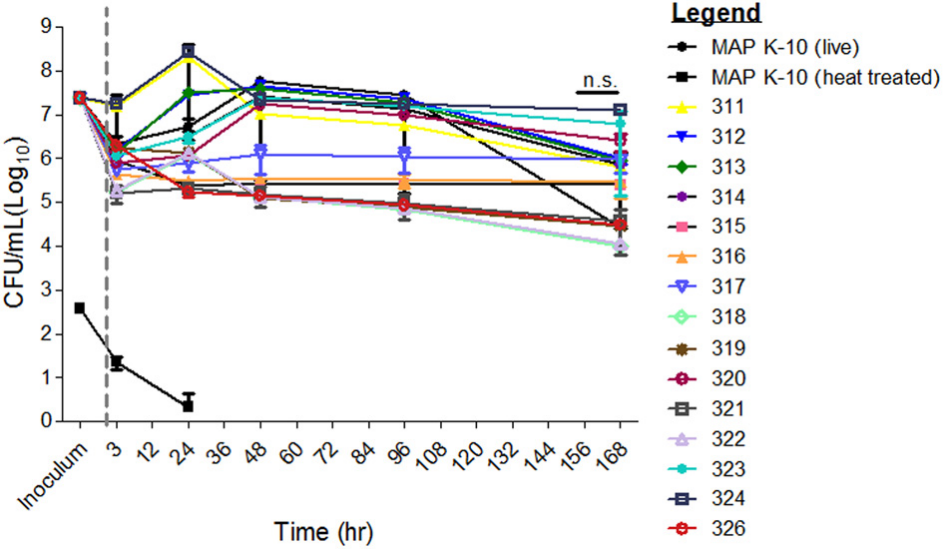
**Survival of MAP mutants in a primary cell *in vitro* bovine MDM model**. All MAP mutant inoculums were normalized to that from MAP strain K-10. Bovine MDMs were incubated at 3, 24, 48, 96, and 168 h. and subsequently lysed for MAP CFU culture. MAP mutant CFU counts did not differ significantly from the control, MAP strain K-10. Error bars represents standard error from the mean (s.e.m). Experiment represents data from 2 cows from the University of Minnesota. All time points were conducted in triplicate. n.s. is abbreviated for not significant. Gray line separates inoculum from the infection time points.

Survival of MAP mutants was performed in two stages. First, a direct comparison of all mutants against K-10 was performed. Second, a slope trend was determined using a mixed linear model approach. Table 1 lists mutant identification, transposon insertion location and method. Survival of MAP mutants was determined at 3, 24, 48, 96, and 168 h p.i. in a well-defined bovine MDM model (Figure 1). Due to varying CFUs in bacterial inoculums, all strains were normalized to MAP K-10 inoculum. All MAP strains, including MAP K-10, showed similar CFU counts throughout the course of the study (Figure 1). Statistical analysis of mutant CFU results compared to MAP K-10 showed no significant (*P* > 0.05) differences at any time point.

**Table 1:** **MAP mutant identification, strain information, and slope estimates**.

**JDIP core identification**	**Strain information**				**Slope estimate^a^**
	**Name**	**Background**	**Location**	**Method**	
311	Δ 482-3	43432-02 (Goat)	MAP0482 and MAP0483	Site directed deletion (McGarvey, unpublished)	−0.0421
312	K10-Δ *relA*	K-10 (Cattle)	*relA*/deletion of the region between 732th bp and 1604th bp of the gene	Phage/site directed (Park et al., 2008)	0.0270
313	K10-Δ *pknG*	K-10	*pknG*/deletion of the region between 406th bp and 2142th bp of the gene		0.0294
314	K10-Δ *lrs2*	K-10	*lsr2*/deletion of the region between 9th bp and 319th bp of the gene		0.0336
315	STM68 (GPM401)	K-10	Internal to MAP1566, insertion is located near the 3' end	Transposon mutagenesis; phAE94 to introduce Tn5370 (Liveneh et al., 2005)	−0.0265
316	2E11 (GPM402)	K-10	Intergenic, 137 bp upstream from *fadE5*, between MAP3695 and FadE5	Transposon mutagenesis; phAE94 to introduce Tn5367 (Liveneh et al., 2005)	−0.0211
317	22F4 (GPM403)	K-10	Internal to *lsr2* (MAP0460)		0.0003
318	40A9 (GPM404)	K-10	Intergenic, between MAP0282c and MAP0283c		−0.018
319	30H9 (GPM405)	K-10	Internal to MAP1566		−0.0146
320	3H4 (GPM406)	K-10	Intergenic, between MAP2296c and MAP2297c		0.0186
321	4H2 (GPM407)	K-10	Intergenic between MAP1150c and MAP1151c		−0.0130
322	WAg906 (TM58)	989 (C strain; Type II)	MAP1566	Transposon mutagenesis (Tn5367) (Cavaignac et al., 2000)	0.0038
323	WAg915	K-10	*ppiA* (MAP0011) inactivated by allelic exchange	Site directed mutagenesis using a phage vector	0.0236
324	*kdpC*	ATCC 19698	MAP0997c*kdpC*	Transposon mutagenesis (Tn5367) (Shin et al., 2006)	−0.0025
325	*lipN*	K-10	Δ *lipN*	Homologous recombination (Wu et al., 2007)	0.0043
326	*umaA1*	ATCC 19698	MAP3963 *umaA1*	Transposon mutagenesis (Tn5367) (Shin et al., 2006)	−0.0284
329	*fabG2_2*	ATCC 19698	MAP2408c *fabG2_2*	Transposon mutagenesis (Tn 5367) (Wu et al., 2007)	0.0198

a *Slope-estimates based-off of data recorded from the University of Minnesota and the University of Wisconsin-Madison*.

In as much as, direct comparisons over the entire infection cycle did not capture differences, a mixed model approach was applied to identify slope trends between strains. Attenuation was determined by slope-value for each MAP strain (Table 1). Mutants that showed a negative slope were identified as attenuated in the MDM model. MAP mutant strains selected to proceed to Phase II studies were required to either show a decaying trend (i.e., negative slope-estimate) or induced apoptosis in PBMCs as described (Kabara and Coussens, 2012). Mutant strains 312–314 and 322–326 showed positive slopes; therefore, these mutants were excluded from the Phase II study (Table 1). Mutant strains 315, 316, 318, 319, and 321 showed negative slopes ranging from −0.0265 to −0.018 (Table 1). The inclusion of strains 320 and 329 (referred to as Mutant 219 in Kabara and Coussens, 2012) were based on apoptosis collected from another JDIP core laboratory (Kabara and Coussens, 2012). Based on these analyses, JDIP 315–321 and 329 were selected for further evaluation in Phase II studies.

## Discussion

Johne's disease vaccination was first described in 1926 using a live strain of MAP mixed with olive oil, paraffin, and pumice powder as an adjuvant (Valle and Rinjard, 1926). Johne's disease vaccine strategies have expanded and diversified due to the public availability of the MAP genome and gene regulation studies (Li et al., 2005; Bannantine and Talaat, 2010; Janagama et al., 2010; Chen et al., 2012). Currently, potential vaccine models for Johne's disease include heat-killed bacilli, live attenuated mutants, recombinant MAP proteins, and MAP genes integrated into viruses or unrelated bacterial plasmids (Cavaignac et al., 2000; Rosseels and Huygen, 2008; Faisal et al., 2013). However, the only licensed Johne's disease vaccine within the United States, Mycopar– comprised of heat-killed *M. avium* (erroneously labeled as MAP in the literature prior to 1993), and other heat-killed formulations do not provide protection against infection and is fraught with negative immunization sequelae (Chiodini, 1993; Ghadiali et al., 2004; Eppleston and Windsor, 2007; Paustian et al., 2008; Patton, 2011; Stringer et al., 2013). The use of Mycopar is restricted to herds with high MAP prevalence in order to reduce MAP shedding and/or delay disease onset (Usda-Aphis, 2010). In order to achieve Johne's disease eradication it is critical that a vaccine that elicits protection be included with strong, standardized management protocols. In a series of three studies (Phase I–III), MAP mutants submitted to JDIP were evaluated for vaccine candidacy. This study (Phase I) evaluated MAP mutant attenuation in an *in vitro* MDM model.

Screening of MAP mutants against strain K-10 was conducted in a well-defined *in vitro* bovine MDM model to define attenuation. Previous studies have shown that bovine MDMs serve as an efficient mechanism for rapid analysis of potential vaccine candidates (Langelaar et al., 2005; Scandurra et al., 2009, 2010). CFU data were first analyzed for the full 7-day period in an attempt to identify trends in attenuation. Since most drop in CFUs were restricted to an earlier infection time point a slope based approach was applied where each mutant was compared against others for 48-h post infection. Mutants with highest slopes (indicating higher rates of intracellular incompetence) were selected as likely attenuated strains for further screening in a mouse model for immunogenicity. While slope values and percent cell apoptosis predicted attenuation, it did not correlate with protection as determined by the results of Phase II and Phase III studies (Bannantine et al., 2014; Hines et al., 2014). For example, in Phase II trials MAP mutants 316–318 showed the highest bacterial burden in cultured tissues (spleen and liver) (Bannantine et al., 2014). Although MAP strain 315 outperformed all other mutants tested in the murine model, the undiluted Silirum vaccine offered the greatest protection as determined by decreased counts of bacterial burden in tissues (Bannantine et al., 2014). Poor protection against standard type infection using MAP mutant strains were also demonstrated in a goat challenge model (Hines et al., 2014). The MAP mutant strain 329 performed the best in the goat model as it reduced bacterial shedding, lowered bacterial burden, and decreased tissue lesion scores (Hines et al., 2014). However, like the murine study, strain 329 failed to outperform the Silirum vaccine. Together these data highlight the limitations of overextending the bovine MDM attenuation model as a proficient tool for initial screening of MAP mutants to predict vaccine candidacy i.e., immunogenicity or ability to prevent infection.

Although traditional vaccine strategies largely rely on attenuation either by heat inactivation or mutation, this strategy appears to be unsuccessful for mycobacteria. For example, *Mycobacterium BCG*, the only licensed vaccine against human tuberculosis (TB), contains multiple deletions but has variable efficacy in adults and fails to protect against pulmonary TB (Behr, 2002; Lewis et al., 2003; Kaufmann, 2013). Current vaccine designs for TB include improvements upon BCG by the addition of foreign genes, such as listeriolysin, that forces the pathogen to be presented to the immune system via an unnatural mechanism (Grode et al., 2005). It is likely that Johne's disease vaccines will need to utilize similar strategies to elicit immunity. Promising Johne's disease vaccine candidates utilizing unconventional methods include those that present MAP antigens in a Salmonella backbone, recombinant MAP proteins and DNA constructs (Kadam et al., 2009; Santema et al., 2009, 2011; Chandra et al., 2012; Faisal et al., 2013).

Despite limitations in the MDM model to predict good vaccine candidates instead of simply measuring attenuation, screened MAP mutants may direct future design of vaccines. For instance, MAP mutants will provide critical clues as to how MAP is processed by the immune system. Host and MAP mutant transcriptomes will provide information pertaining to conserved pathways that should be considered during vaccine design (Lamont et al., 2013).

## Conclusions

Phase I showed that attenuation of *in-vitro* growth rates in MDM do not correlate with ability to prevent infection in a relevant animal model of infection. These results highlight one of the limitations of the widely used bovine MDM model for vaccine candidate identification based on an attenuation phenotype alone. Future studies should consider alternate vaccine candidate screens and include multiple readouts including—antigen processing and presentation, efficiency of modulating subcellular processing events and ability of vaccine candidates to induce specific cytokines, in addition to the survival trait.

## Author contributions

Elise A. Lamont was responsible for performing invasion assays in MDMs, primary analysis and interpretation, and writing of the manuscript. Srinand Sreevatsan, Adel Talaat, Paul M. Coussens, John P. Bannantine and Vivek Kapur were responsible for generating the ideas, obtaining funding, and project design. Yrjo T. Grohn was the JDIP Core statistician and responsible for calculating and interpreting slope data. Ling-ling Li and Robab Katani were responsible for culture, coding, and shipment of MAP mutant strains.

### Conflict of interest statement

The authors declare that the research was conducted in the absence of any commercial or financial relationships that could be construed as a potential conflict of interest.
